# Cultural Competence in the nursing, dentistry, and medicine professional curricula: a qualitative review

**DOI:** 10.1186/s12909-022-03743-7

**Published:** 2022-09-20

**Authors:** Maura Klenner, Rodrigo Mariño, Patricia Pineda, Gerardo Espinoza, Carlos Zaror

**Affiliations:** 1 Deparment of Language, Literature and Communication, Faculty of Education, Humanities and Social Sciences, Universidad de La Frontera, Temuco, Chile; 2 Melbourne Dental School, University of Melbourne, Melbourne, Australia; 3 Department of Pediatric Dentistry and Orthodontics, Faculty of Dentistry, Universidad de La Frontera, Temuco, Chile; 4 Department of Public Health, Faculty of Medicine, Universidad de La Frontera, Temuco, Chile; 5 Present Address: Center for Research in Epidemiology, Economics and Oral Public Health (CIEESPO), Faculty of Dentistry, Universidad de La Frontera, Temuco, Chile

**Keywords:** Cultural competence, Healthcare, Curriculum, Course syllabus, Teaching–learning methodologies

## Abstract

**Background:**

Cultural competence development in the formative process of healthcare professionals is crucial for the provision of culturally appropriate health care. This educational issue is highly relevant in the growing multicultural composition of southern Chile. The objective of this study was to examine how the healthcare professions curricula at the Universidad de La Frontera, in La Araucanía Region, prepares future professionals to respond to patients' cultural needs.

**Method:**

A sequential transformative mixed methods design composed of two phases was carried out. Phase 1 reviewed all printed material and documentation to explore content that developed cross-cultural skills and competencies in the curricula. In Phase 2 semi-structured interviews were conducted with academics with responsibilities for the development of the curriculum in each career, to detect how academics envisage the incorporation of cultural competence in the curricula.

**Results:**

Regarding curricular contents, findings indicated that the healthcare professions curricula at The Universidad de La Frontera have similar approaches to the inclusion of CCT in subjects’ syllabuses, with inclusion of the different CCT, particularly in the Dental and Medical curricula. However, this coverage showed significant variations in the undergraduate healthcare curricula. The analysis revealed that themes around the Ethics and human values for professional practice; the Psychosocial and cultural determinants of health; the Relationship health-family-community, and to a lesser extent, the Clinician-patient relationship were well covered in the courses. On the other hand, Inequalities in health was the theme with the least contact time in all three courses.

Academics called for a better organisation of the inclusion of CCT in the curricula. They also highlighted the challenges of maintaining the dominant paradigm underlying healthcare models, practices, and orientations within the academic staff and health discipline.

**Conclusion:**

Curricula contents findings indicate that the healthcare professions curricula at Universidad de La Frontera have similar approaches to the inclusion of CCT in subjects’ syllabuses. However, its depth of coverage allows for improvements. The systematization of CCT and teaching–learning methodologies in healthcare professions curricula is necessary to develop formative processes that allow future professionals to be aware of and respectful with patients’ cultural characteristics and needs.

**Supplementary Information:**

The online version contains supplementary material available at 10.1186/s12909-022-03743-7.

## Introduction

Cultural competence (CC) in health care is defined as "a process in which the health care provider continually strives to achieve the ability to work effectively within the cultural context of a patient." This includes five key interdependent elements: cultural awareness, cultural knowledge, cultural ability, cultural encounters, and cultural desire [1] Campinha-Bacote developed her model in 1969, deriving her work from several authors in the multicultural field of healthcare [2–4]. The CC framework for this study is based on the synthesis of the culturally competent care models of Helman [5] and Campinha-Bacote [6].

More recently, the concept of cultural safety has been introduced. Cultural safety is defined as ‘the ongoing critical reflection of health practitioner knowledge, skills, attitudes, practising behaviours and power differentials in delivering care that is safe, accessible and responsive healthcare free from racism' [7, 8]. Cultural safety is now part of accreditation standards in many countries in the understanding that there is no clinical safety without cultural safety [8].

Cultural competence and safety are considered as a continuous process consisting of five constructs [1, 6]. These five constructs have an independent relationship; however, the model is dynamic, therefore, any improvement in one of them will result in improvements in all five constructs. Furthermore, the model does not infer a static level of achievement [9], it represents an ongoing reflective process focused on critical self-reflection on one’s cultural bias, privileges, and power, rather than on components of the culture of others [9].

The model of cultural competence and safety focuses on the process by which healthcare professionals develop cultural awareness, knowledge and skills that lead to changing attitudes, valuing diversity, and understanding their own cultural biases. Therefore, this process requires providers to be truly culturally competent, with prior cultural encounters, and to possess cultural awareness, knowledge, and skills that enable effective cross-cultural communication [1, 2, 6]. This process enhances the ability of healthcare providers to provide culturally safe care in a multicultural healthcare setting [9].

In addition, the model emphasizes that the healthcare encounter should include effective cross-cultural communication. This process is based primarily on the cultural desire that requires providers to be truly culturally competent, in addition to having had cultural encounters and possessing cultural awareness, knowledge, and skills [1, 6, 9].

The interaction between healthcare professionals and patients in the context of a clinical encounter could be explained using the interactive model [10]. According to this model, a healthcare encounter is influenced by the culture of two contexts: 1) the external context that refers to the culture of health care and health education and 2) the internal context that consists of culture and health, and the influences of the healthcare professional and the patient. The success of the outcome of the encounter would depend on the level of interaction between the healthcare provider and the patient and the way the patient presents the symptoms of the disease and how the health care provider receives and interprets this presentation in an appropriate diagnosis and plan of adequate treatment. This treatment plan has to be agreed on and accepted by the patient to achieve best results. Successful encounters between healthcare professionals and patients result in effective communication, patient satisfaction, patient compliance, and optimal health outcome [5].

Chile, like most present-day countries, has culturally diverse societies composed of numerous cultural groups such as First Nation people, recent immigrants (e.g., from other Latin American countries), previous immigrants (e.g., Spaniards, Italians, Germans, etc.), who coexist within a culture more predominantly influenced by Spanish colonialism.

It is estimated that the foreign-born population in Chile increased by a 12.4% between 2018 and 2020 [11]. The majority of the foreign-born population come from Venezuela (30,7%), Perú (16,3%), Haiti (12,5%), Colombia (11,4%) and Bolivia (8,5%) [11].

Many immigrants and indigenous people retain native languages and traditions, generating not only ethnic or linguistic diversity, but also cultural differences [12]. Each culture has unique beliefs, practices, and expectations about health care, which makes CC of great importance to health professionals [12]. Therefore, it is inevitable that future health professionals will work with patients from different cultures and must cross cultural boundaries to provide care that is culturally sensitive and responsive to the multicultural base of the population.

Transcultural skills have been regarded as crucial for healthcare professionals who work in inter or multicultural scenarios. Cultural and linguistic mismatches between healthcare professionals and patients have been highlighted as one important source of difficulty in conducting proper medical care [13, 14]. In contexts of cultural diversity, successful health outcomes have been linked to professionals who are sensitive to their patients’ cultural background and characteristics. Thus, providing supporting evidence that it is necessary to move into a more culturally competent provision of health services. This fact reveals the importance of providing cross-cultural training in healthcare curricula.

Although CC is a process, its acquisition and development must begin during the education of the health professional [14, 15]. Without these professional resources, serious cultural disagreements can potentially occur, leading to the creation of barriers in the provision and delivery of oral health care and the subsequent loss of the relationship between health professionals and these patients [12, 15].

To achieve CC, it is essential that healthcare profession students, receive training that promotes the knowledge necessary to understand culturally influenced health behaviours, as well as acquiring the ability to communicate effectively with patients from cultures other than them [16–18].

The aim of this study was to examine how the healthcare professions curriculum (Dentistry, Medicine and Nursing) at The Universidad de La Frontera, Temuco, Chile, prepares future professionals for efficient and safe management of patients' cultural diversity needs. More specifically, the study will: a) gain information about the content and development of cross-cultural skills and competencies; and b) describe how the academics involved in the planning and organizing of the curriculum of these programs envisage the incorporation of cultural competence into the study plan.

This study will provide information for the development of strategies to improve healthcare profession students’ capacity for intercultural communications. This information will also contribute to better relationships between healthcare professionals and their patients. In turn, this will translate into better treatment outcomes, and patient satisfaction. Methodological strategies for cross-cultural education would respond to the educational needs detected by the study to expand cultural competencies among students of Medicine, Dentistry and Nursing at The Universidad de La Frontera.

## Method

**Fig. 1:**
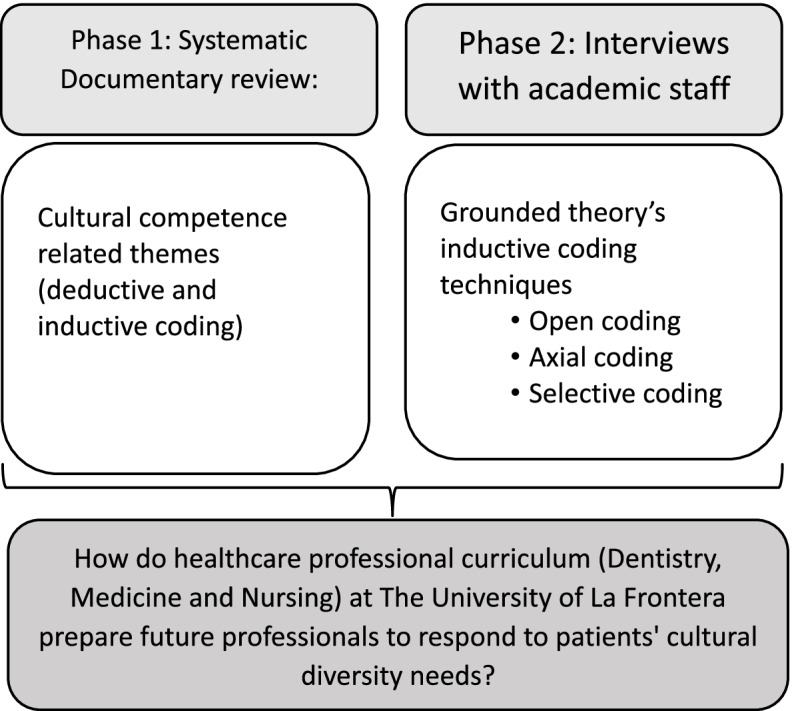
Sequential transformative mixed methods design

To achieve the objectives of this study, the project adopted a sequential transformative mixed methods design composed of two phases [19], as shown in Fig. 1. This design was selected because qualitative data for Phase 1 was collected and integrated with data collected in Phase 2 in the interpretation stage.

### Phase 1: Systematic documentary review

This phase’s objective was to obtain information about the curricula contents for the development of cross-cultural skills and competencies that prepare future healthcare professionals for the appropriate management of patients with diverse cultural backgrounds. To achieve this phase’s objective, a systematic documents review was conducted in order to examine how cross-cultural education has been incorporated into the curricula of three healthcare courses: Dentistry (6 Years), Medicine (7 Years), and Nursing (5 Years).

The analysis was conducted to analyse the subject syllabuses from each of the three courses. Within each course, subject syllabuses were collected and analysed considering the representation of cultural competence related themes (CCT). The evaluation of the presence of transcultural content in subjects’ syllabuses was based on six pre-determined themes [14, 20], following a deductive coding. The analysis of the data was conducted by the research team, directed by the researcher with more experience in content analysis. Pre-determined CC themes were identified in the syllabuses:

- Psychosocial and cultural determinants of health
- Concepts of cultural diversity
- Beliefs, behaviours and expectations related to health and illness
- Inequalities in health
- The clinician-patient relationship
- Indigenous people concepts of health and disease

Inductive coding was also carried out to determine whether there were any additional CCT themes that were not considered in prior research that were relevant to this study.

The initial analysis was presented to the other researchers, who audited the analysis, reaching stability in the findings. Codes were created and patterns were found in course syllabuses indicating that other themes regarding CC were present. The analysis was presented to the other researchers, who discussed it in light of existing literature. Key CC concepts and themes are described in Supplemental Table 1.

**Table 1:** Cultural Competence related Themes by Depth of coverage at the Dentistry study program

Cultural Competence Theme	**Depth of coverage**			
	Consistent	Intermediate	Emerging	Total
Psychosocial and cultural determinants of health	9.1%	20.0%	20.0%	**49.1%**
Concepts of cultural diversity	3.6%	1.8%	3.6%	9.1%
Beliefs, behaviours and expectations related to health and illness	0.0%	1.8%	0.0%	1.8%
Inequalities in health	0.0%	0.0%	0.0%	0.0%
The clinician-patient relationship	9.1%	16.4%	10.9%	**36.4%**
Indigenous people concepts of health and disease	0.0%	1.8%	1.8%	3.6%
Relationship health-family-community	7.3%	16.4%	5. 50%	29.1%
Ethics and human values for professional practice	16.4%	21.8%	18.2%	**56.4%**
Intercultural communication	3.6%	1.8%	3.6%	9.1%

All themes were analysed in relation to how deeply established and developed they were in the syllabuses, aspect which will be referred to as depth of coverage. Four categories of syllabus were identified:

- **Consistent** syllabuses are those in which the approach to elements that allow the development of CCT in various sections of the subjects’ syllabus is observed, demonstrating coherence between competencies, learning outcomes, contents, methodologies, and bibliography.
- **Intermediate** syllabuses contain elements that allow the development of CCT in some sections of the subject’s syllabus, however, there is no internal coherence of these elements in the subject program.
- **Emerging** syllabuses are those in which the presence of elements that allow the development of CCT is observed, however, these are not observed in all sections of the syllabus, and do not follow a solid organization.
- **Not observed** correspond to those in which none of the CCT were found.

#### Phase 2. Interviews with academic staff

Sixty-one academics from the subjects assessed in Phase 1 were invited to take part in individual face-to-face interviews. For some of the subjects, academics were in teaching teams, so they nominated one of them to represent the team to provide an account of their methodological approach to the subject. In other cases, one academic was in charge of more than one subject. For this reason, in Medicine, a professor who was in charge of four subjects in that course took part (See Supplemental Table 2). A total of 7 academics participated in this study.

**Table 2:** Cultural Competence related Themes by depth of coverage at the Nursing study program

**Cultural Competence Theme**	**Depth of coverage**			
	Consistent	Intermediate	Emerging	Total
Psychosocial and cultural determinants of health	8.0%	20.0%	0.0%	**28.**0%
Concepts of cultural diversity	0.0%	8.0%	0.0%	8.0%
Beliefs, behaviours and expectations related to health and illness	0.0%	0.0%	0.0%	0.0%
Inequalities in health	0.0%	0.0%	0.0%	0.0%
The clinician-patient relationship	16.0%	12.0%	0.0%	**28.0%**
Indigenous people concepts of health and disease	4.0%	12.0%	4.0%	20.0%
Relationship health-family-community	16.0%	32.0%	40.0%	**88.**0%
Ethics and human values for professional practice	16.0%	36.0%	12.0%	**64.**0%
Intercultural communication	0.0%	4.0%	0.0%	4.0%

These interviews were aimed at obtaining further information, and clarification of the information collected. Three general themes were explored in the interviews:

- Importance attributed by the interviewee to the teaching of cross-cultural skills in the courses’ curriculum and in professional performance.
- Description of teaching–learning, methodologies (resources, strategies, activities, evaluation) for the management of content and cross-cultural skills in the identified subjects.
- Challenges and barriers faced in teaching cross-cultural content and skills.

Prior to the interviews, the researcher provided potential participants with a copy of the Plain Language Statement, verbally clarified the aims of the study, and explained the interview and analysis process, data security, and the individual’s right to withdraw. Those who volunteered to participate were requested to provide verbal consent to participate and to send a signed consent to the interviewer via e-mail. All interviews were video and audio recorded and transcribed for further analysis. Additionally, the interviewer scribed general notes of the responses throughout the interview. Notes and transcriptions were reviewed by the researcher conducting the interviews, the project leader, and another member of the research team to ensure accuracy and consistency. Transcripts were eliminated of any identifying information. Pseudonyms were assigned to each participant and used in transcripts and reports.

This phase adopted a content analysis method, following Grounded Theory’s inductive coding techniques (yet not following a Grounded Theory Design) [21]. Interviews were analysed through the constant comparative method, following its specific stages of open, axial, and selective coding. Open coding: initial conceptual categories were formulated in relation to academics’ experiences regarding their development of CCT in the curriculum. Axial coding: the data were regrouped, and the initial conceptual categories were related to their subcategories (causal, intervening, contextual conditions) that affected the object of study. Selective coding: the understanding of the categories was integrated and refined around a central category, obtaining an integrating scheme to account for the relationships among them.

The scheme was validated by iteration with the data. However, it was not possible to reach theoretical saturation due to the number of interviews conducted. The content analysis was performed using the ATLAS.ti 8.4 software.

## Results

### Phase 1: Systematic documentary review

A systematic documentary review was carried out on the three selected healthcare professional programs. The documents analysed corresponded to the University’s Professional Training Policy, the course study plans, and 144 subject syllabuses (55 in Dentistry; 25 in Nursing; and 64 in Medicine). Five elective subjects were not included in this analysis: two in Dentistry and three in Nursing.

### Phase 1. Cultural competence related themes (CCT)

CCT included 6 predetermined themes and 3 themes that emerged from the inductive analysis. Emergent themes identified were:

- Relationship health-family-community: It refers to the role of family and community in the prevention of illness, and the understanding of how family and community culture interfere in healthcare prevention and treatment.
- Ethics and human values for professional practice: It refers to the ethical practices that healthcare professionals should incorporate into their professional activities, such as respectful attitudes toward their patients’ cultural background and practices.
- Intercultural communication: The abilities, skills, and knowledge that allow the healthcare professional to effectively interact with patients from different cultural backgrounds.

Tables 1, 2and 3 show the percentage of subjects’ syllabuses that contain each of the cultural competence related themes by professional course. Two themes (Psychosocial and cultural determinants of health) and (Ethics and human values for professional practice) were the most repeated across the three courses.

In the case of Dentistry, Ethics and human values for professional practice; Psychosocial and cultural determinants of health; and the Clinician-patient relationship were the most common themes in the subjects’ syllabuses. The theme Inequalities in health was not observed in the Dental syllabuses. Table 1 presents the distribution of CCT by depth of coverage.

In the case of Nursing, Relationship health-family-community; Ethics and human values for professional practice; Psychosocial and cultural determinants of health and Clinician-patient relationship were the most common themes of the syllabuses. The themes Beliefs, behaviours and expectations related to Health and illness and Inequalities in health were not observed in any of the subjects’ syllabuses of the nursing course. Table 2 presents the distribution of CCT by depth of coverage in the Nursing study program.

In the case of Medicine, themes Ethics and human values for professional practice; Psychosocial and cultural determinants of health, and Relationship health-family-community were the most found in the syllabuses analysed in the medical courses. Table 3 presents the distribution of CCT by depth of coverage in the medical course.

**Table 3:** Cultural Competence related Themes by depth of coverage at the Medicine study program

**Cultural Competence Theme**	**Depth of coverage**			
	Consistent	Intermediate	Emerging	Total
Psychosocial and cultural determinants of health	7.8%	12.5%	12.5%	**32.8%**
Concepts of cultural diversity	4.7%	7.8%	9.4%	21.9%
Beliefs, behaviours and expectations related to health and illness	1.6%	0.0%	3.1%	4.7%
Inequalities in health	1.6%	0.0%	0.0%	1.6%
The clinician-patient relationship	7.8%	7.8%	4.7%	20.3%
Indigenous people concepts of health and disease	1.6%	9.4%	0.0%	10.9%
Relationship health-family-community	7.8%	**9.4%**	6.3%	**23.4%**
Ethics and human values for professional practice	10.9%	15.6%	**25.0%**	**51.6%**
Intercultural communication	4.7%	0.0%	0.0%	4.7%

### Phase 2: Information retrieved from the interviews with academic staff

**Fig. 2:**
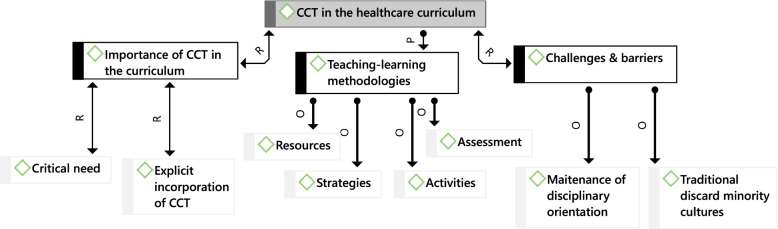
Academics report on the incorporation of CCT in the healthcare curriculum

Seven academic staff consented to participate in this phase of the study. They reported their opinions on the three main topics, as illustrated in Fig. 2:

#### Importance attributed to CCT

Academics agreed on two aspects of this topic: a) the critical need for including CCT in the healthcare professions curricula and, b) the need to address these topics more explicitly in subjects, through specific teaching–learning activities. These activities were mainly related to of the need to incorporate alternative conceptions of healthcare provided by local indigenous and immigrant cultures to offer more assertive treatments to patients. Additionally, academics observed a growing multicultural representation in their daily professional practices in both urban and rural healthcare centres.

#### Teaching–learning methodologies

**Fig. 3:**
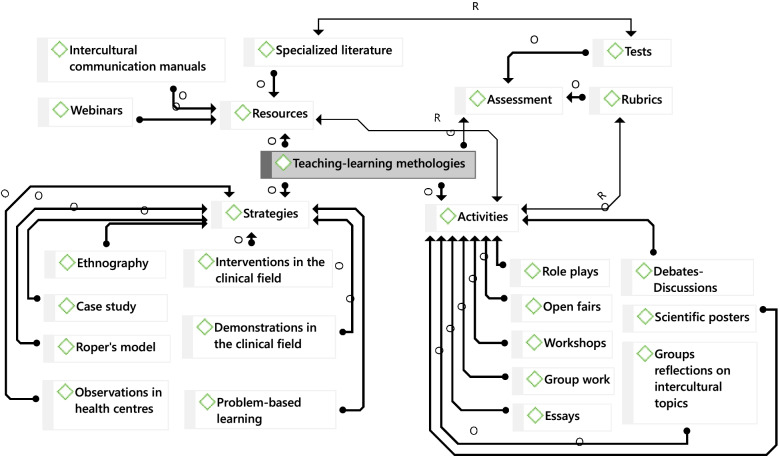
Teaching–learning activities reported by academic staff

Although the academics agreed that the incorporation of CCT needed more explicit consideration, they identified a set of teaching–learning methodologies they have included in their subjects for the management of content and CC and safety skills. Figure 3 presents a classification of the activities that have already been incorporated into the subjects. These methodologies can be divided into four categories: resources; strategies; activities; and assessment strategies.

In relation to resources, academics reported the use of specialized literature, webinars, and intercultural communication manuals. They relied mainly on the use of specialized literature as a basis for their classes. This literature included specialized references on varied cultural practices of local cultures, as well as immigrant cultures. Specialized literature also included references regarding different theoretical perspectives on cultural related themes.

Webinars have been widely used during the COVID-19 pandemic as a resource that allows students to have access to first-hand contact with specialists located in different jurisdictions. Intercultural communication manuals and resources have been designed by academics and are specific to the local context in relation to immigration [22].

Strategies mentioned by the academics in relation to the development of transcultural themes were some types of research methods, such as ethnography, case-studies, and problem-based learning, in which students must analyse clinical scenarios and patients from a transcultural perspective. Furthermore, they also reported some types of practice-oriented strategies, such as Roper’s model [23], observations in health centres, and clinical demonstrations and interventions, which allow students to work on the connection between knowing and knowing how*.*

Activities mentioned by the academics were: the use of role plays; open showcases about intercultural topics; workshops; group work; writing essays; class debates and discussions; the elaboration of scientific posters; and group reflections on intercultural topics. These activities were related to the materials provided by the lecturer in each subject.

Academics reported two main assessment strategies: tests associated with conceptual and theoretical knowledge about transcultural topics; and the use of rubrics associated with some of these activities, whenever they constituted an evaluation activity. Academics reported that the use of rubrics allowed them to assess whether students had achieved and were able to demonstrate specific competencies related to transcultural communication.

Challenges and barriers encountered by participants when teaching cross-cultural content and skills were a) the maintenance of a disciplinary orientation of healthcare professions programs; and b) the traditional discarding of minority cultures’ knowledge and practices related to healthcare. In regard to the maintenance of a disciplinary orientation of healthcare professional programs, academics reported that traditional teaching approaches based on disciplinary knowledge were more valued than approaches based on the development of competencies, in this case transcultural competence. This resulted in students and newly graduated health professionals reproducing those judgements. The change of teaching styles and judgements was considered one of the main challenges to the implementation of innovated curricula and teaching strategies.

Academics also identified some resistance to change in their academic colleagues, which they attributed to personal world views and value systems, as well as to the perceived expectations from their profession and the traditional lack of appreciation of local indigenous cultures. Participants agreed that although these cultural perspectives are becoming more acknowledged in the undergraduate curricula, particularly in recent years, there is still a wide range of subjects which do not include them. Academics commented that the inclusion of CCT has been an extremely controversial topic during curricular reviews. For example, academics commented on the polarization of the staff between those academics with a disciplinary focus, and those with a more integrative view of the patients’ needs.

Experiences of culturally oriented intervention programs in rural health centres were reported as a way to demonstrate the importance attributed to the inclusion of these themes, not only in the curricula, but also as part of new study lines among academics.

## Discussion

There is an increased recognition of the importance and understanding that cultural competence and safety is a learned process. Consequently, a systematic documents review was conducted to examine how cross-cultural education has been incorporated into the curricula of three healthcare courses (e.g., Dentistry, Medicine, and Nursing). This study also reviewed the cultural challenges faced when implementing a culturally safe healthcare curricula and the academics perception of the coverage of cultural aspects in the curriculum of healthcare courses. The literature indicates that compared to medicine and nursing, information on cross-cultural dental education is relatively limited [1, 3, 6, 16, 20, 24–30].

Regarding curricular contents, findings indicated that the healthcare professions curricula reviewed at The Universidad de La Frontera, have similar approaches to the inclusion of CCT in subjects’ syllabuses, with inclusion of the different CCT, particularly in the Dental and Medical curricula. However, this coverage showed significant variations in the undergraduate healthcare curricula. The analysis revealed that themes around the *Ethics* and human values for professional practice; the Psychosocial and cultural determinants of health; the Relationship health-family-community, and to a lesser extent, the Clinician-patient relationship were well covered in the courses.

On the other hand, areas which were poorly covered included Beliefs, behaviours and expectations related to health and illness; and Indigenous people concepts of health and disease. In particular, the Inequalities in health theme, was covered marginally in the Medical curriculum only. Similar cultural safety content (shortage and coverage) has been noted elsewhere [14, 20, 31]. However, its depth of coverage allows for improvements. Although in all three courses, most of the themes were present, they covered with intermediate or incipient depths. Thus, teaching of cultural competence remained fragmented and inadequate [32].

Evidence from the interviews with academics supported these conclusions. Academics called for a better organisation of the inclusion of CCT in the curricula. That is, an improved organization of themes within subjects in relation to how they are included in their different aspects (i.e., competencies, learning outcomes, contents, methodologies –teaching/learning, evaluation – and bibliography). Although academics described various teaching–learning methodologies used for the teaching of CCT, they also recognized that there is an urgent need to include these CCT related methodologies in a more explicit and consistent manner in the curricula.

Academics also highlighted the challenges generated by the maintenance of a disciplinary orientation within the academic staff and health discipline, which focuses on a biomedical model of healthcare, and a lack of appreciation and knowledge of traditional model of healthcare practices of indigenous cultures and of other minority groups within the national healthcare system. These findings echoed those of Farías-Cancino et. al [33]. who highlighted that the development of CC in the curriculum should follow a purposed method, which would allow the inclusion of CCT in a systematic manner, and that consisted on the selection of a theoretical CC model, the construction of a CC definition contextualized to the tertiary education institution, and the clarification of CC levels students are expected to achieve, in order to incorporate them in subject syllabuses through teaching–learning and evaluation methodologies.

As such, it is possible that the current approach towards CCT in healthcare professions training might lead to an incomplete or biased understandings of the type of relation professionals should establish with their patients. These might become less patient-centred, less positive, and prone to poor communication and cultural discrimination [9].

Although this study provided valuable insights into the cultural competence training of healthcare profession students, it is not without limitations. The most obvious limitation was the fact that the data were restricted to only one institution, the only state university in La Araucanía Region, which might result in conclusions that are not applicable to all Chilean healthcare professions curricula. Additionally, the study relied on self-reported data from motivated academic, and there is the probability of social desirability bias. However, considering these limitations, we believe that the current approach was adequate, particularly given the exploratory nature of the study.

Thus, this inquiry is only in its initial stages. Notwithstanding, information from this study is significant because it is the first Chilean study to document the extent in which CC content is covered, and to compare how those contents are covered in the undergraduate curricula of healthcare professions. The high proportion of First Nation people living in the Region where this university is located, highlights the importance of health professionals having the appropriate cultural competences to provide a cultural safe practice.

Furthermore, present findings become even more relevant due to the growing interest and need to include CC content in healthcare professions curricula. In fact, the United Nations has recognized culture as a causal agent of sustainability and integrated it into the SDG goals [34]. This need will only continue to increase in countries like Chile, due to the changing sociodemographic characteristics of the population [35]. Having those skills would endure a more positive health outcome [5]. Additionally, this information is crucial to address current standards and expectations of the National Accreditation Commission [36]. This project will also ensure that priorities established in the Chilean legislature (Law Nº 20.584), which recognise the protection of health care related rights, welfare, dignity, and duties of persons, are achieved.

A better perception of cultural competencies and intercultural safety in the dental, medical, and nursing curricula provides an understanding of their success in addressing the need for cultural competence and safety. Being aware of the current characteristics of healthcare professions study programs provides opportunities to make relevant modifications in order to improve formative processes, and subsequently, local multicultural healthcare scenarios. This will then provide the opportunity to modify the curricula of these courses to improve educational outcomes and in this way respond to the educational needs for the development of intercultural strategies and competencies. Moreover, the study will allow for the establishment of baselines for future comparisons within the same cohorts of students.

Healthcare professionals play an important role in addressing patients’ overall health and well-being, and need to constantly reflect on their skills, expertise, and experience to best support a multicultural patient base. The systematic inclusion of Cultural Competencies related themes and teaching–learning methodologies in healthcare profession curricula is necessary to develop formative processes that allow future professionals to be aware of patients’ cultural characteristics and needs. It is essential that health professionals are able to provide culturally safe healthcare. It is hoped that this study further contributes to creating culturally safe environments and therefore the elimination of cultural and culturally based inequalities in health.

## Supplementary Information

**Additional file 1: Supplemental Table 1.** Key Concepts and contents of cultural competence and transcultural contents.

**Additional file 2: Supplemental Table 2.** Distribution of academic staff invited to participate in the in-depth interview and participants by course.

## Data Availability

The data generated and/or analysed during the current study are not publicly available due to the ethics approval granted on the basis that only researchers involved in the study can access the de-identified data. The minimum retention period is five years from publication. Supporting documents are available upon request to the corresponding author.

## Abbreviations

CCT: Cultural Competence related themes
CC: Cultural competence
