# Supplementary material for: Cultural Competence in the nursing, dentistry, and medicine professional curricula: a qualitative review
Source: BMC Med Educ. 2022 Sep 20;22:686. doi: 10.1186/s12909-022-03743-7 (PMC9485016; doi:10.1186/s12909-022-03743-7)
Supplement: Supplementary file 1 — Additional file 1: Supplemental Table 1. Key Concepts and contents of cultural competence and transcultural contents. [file 12909_2022_3743_MOESM1_ESM.docx]

Supplemental Table 1. Key Concepts and contents of cultural competence and transcultural contents

| Key Concepts | Contents, themes, skills included |
| --- | --- |
| Psychosocial and cultural determinants of health (social determinants) | Definitions and perspectives of health, disease, and disability  The selfhood theoretical perspectives in cases of illness and impairment  Individual experience of illness and decision to seek help  Public and population health concepts Health education, health promotion and advertisements |
| Cultural diversity and safety concepts | Cultural awareness and cultural sensitivity, active reflection on personal attitude and behaviours.  Different cultural beliefs and expectations related to health and illness The importance of adopting a culture sensitive approach to optimize medical outcomes Health services allocation and development according to the needs of culturally diverse patients Ethical responsibilities in the medical practice within the cultural context Attitudes and practices related to death and dying in a post-modern western country compared with other cultures |
| Beliefs, behaviours and expectations related to health and illness | Health belief and medical practice models.  Diversity of health beliefs, using alternative medicine.  Factors which shape individual behaviour and beliefs  Factors which influence healthcare treatment and outcomes within the cultural context  The importance of adapting a culture sensitive approach to optimize medical outcomes.  Health services allocation and development according to the needs of culturally diverse patients.  Attitudes and practices related to death and dying in a post-modern Western country compared with other cultures  Awareness of different cultural beliefs on healthcare practices  Awareness of the current provision of a holistic model of health in relation to transcultural communities  Principles of culturally appropriate health intervention  Valuing patients with different health beliefs and practices |
| Inequalities in health | Equity/inequity in health.  Stigma and impact on health outcomes  Mental health determinants  Social origins of health.  Factors which contribute to health inequalities in relation to Indigenous populations |
| The clinician-patient relationship | Models of doctor–patient relationship. Conceptual models of practice The difference between doctor-centred and patient-centred approach The key features of patient-centred approach Participation in decision-making by patients and families according to their backgrounds The impact of the different settings on patients’ experience of illness Barriers to good care and medical decision-making at the end of life due to current social, religious and cultural factors |
| Transcultural and Aboriginal/Indigenous concept of health and illness | Definitions of culture, ethnicity and community Awareness about transcultural health Aboriginal/Indigenous culture and health The effects of colonization history and segregation and assimilation on Aboriginal/Indigenous health Definitions of health and illness in relation to Aboriginal communities Health status data, burden of diseases and risk factors in Aboriginal/Indigenous communities Migrants’ health issues Immigration patterns Immigrants’ health status and needs Immigration as a risk factor for diseases Culturally appropriate health intervention |
| Relationship health-family-community | The role of family and community in the prevention of illness  Awareness of how family and community culture interfere/mediate in healthcare prevention and treatment |
| Ethics and human values for professional practice | Ethical practices that healthcare professionals should incorporate into their professional activities  Attitudes toward their patients’ cultural background and practices  Informed Consent  Confidentiality  Health Complaints  Regulation of the health care Professions |
| Effective Intercultural communication | Principles, purposes, and the importance of effective communication  Models of communication Patient-centred communication framework for the healthcare consultation  Multidisciplinary team concept in health professionals Types of communication skills Medical interviewing and listening skills Verbal/non-verbal responding skills Interview of a carer about a patient Interview of a patient with a chronic illness.  Giving information to patients Factors which inhibit or enhance the clinician–patient communication  Strategies for working with interpreters |
