# Supplementary material for: Cultural Competence in the nursing, dentistry, and medicine professional curricula: a qualitative review
Source: BMC Med Educ. 2022 Sep 20;22:686. doi: 10.1186/s12909-022-03743-7 (PMC9485016; doi:10.1186/s12909-022-03743-7)
Supplement: Supplementary file 2 — Additional file 2: Supplemental Table 2. Distribution of academic staff invited to participate in the in-depth interview and participants by course. [file 12909_2022_3743_MOESM2_ESM.docx]

Supplemental Table 2. Distribution of academic staff invited to participate in the in-depth interview and participants by course.

| Course | Subject in the course study plan | Staff invited | Participants |
| --- | --- | --- | --- |
| Dentistry | Anthropology | 1 | 1 |
|  | Public Health II and Prevention | 1 | 0 |
|  | Integration Unit II | 1 | 0 |
|  | Basic Clinic III and IV | 8 | 1 |
|  | Psychology II | 1 | 0 |
|  | History of Chilean society and culture | 1 | 0 |
|  | Bioethics | 1 | 0 |
|  | Ethics, Morals and Logics | 1 | 0 |
|  | Family Health | 1 | 1 |
| Nursing | Nursing in Adults II | 14 | 1 |
|  | Nursing in Children and Adolescents | 8 | 1 |
|  | Professional Practicum (Adults) | 2 | 1 |
|  | Professional Practicum (Children) | 5 | 0 |
| Medicine | Module: Administration and Research in Health | 1 | 0 |
|  | Introduction to Medical Studies II | 2 | 0 |
|  | Bioethics | 3 | 0 |
|  | Demographics and Health | 6 | 0 |
|  | Personal and Interpersonal Development I | 1 | 1 |
|  | Personal and Interpersonal Development II |  |  |
|  | Medical Psychology |  |  |
|  | Psychopathology |  |  |
|  | Interdisciplinary Rural Internship Program | 3 | 0 |
